# Dynamic Adipocyte Phosphoproteome Reveals that Akt Directly Regulates mTORC2

**DOI:** 10.1016/j.cmet.2013.04.010

**Published:** 2013-06-04

**Authors:** Sean J. Humphrey, Guang Yang, Pengyi Yang, Daniel J. Fazakerley, Jacqueline Stöckli, Jean Y. Yang, David E. James

**Affiliations:** 1 Diabetes and Obesity Program, Garvan Institute of Medical Research, Darlinghurst, New South Wales 2010, Australia; 2 Biological Mass Spectrometry Unit, Garvan Institute of Medical Research, Darlinghurst, New South Wales 2010, Australia; 3 School of Mathematics and Statistics, University of Sydney, Camperdown, New South Wales 2006, Australia

## Abstract

A major challenge of the post-genomics era is to define the connectivity of protein phosphorylation networks. Here, we quantitatively delineate the insulin signaling network in adipocytes by high-resolution mass spectrometry-based proteomics. These data reveal the complexity of intracellular protein phosphorylation. We identified 37,248 phosphorylation sites on 5,705 proteins in this single-cell type, with approximately 15% responding to insulin. We integrated these large-scale phosphoproteomics data using a machine learning approach to predict physiological substrates of several diverse insulin-regulated kinases. This led to the identification of an Akt substrate, SIN1, a core component of the mTORC2 complex. The phosphorylation of SIN1 by Akt was found to regulate mTORC2 activity in response to growth factors, revealing topological insights into the Akt/mTOR signaling network. The dynamic phosphoproteome described here contains numerous phosphorylation sites on proteins involved in diverse molecular functions and should serve as a useful functional resource for cell biologists.

## Highlights

• MS/MS identified >37,000 phosphorylation sites in adipocytes
• Insulin regulates the phosphoproteome over a wide temporal timescale
• Akt phosphorylates SIN1 on T86 in response to insulin
• SIN1 phosphorylation activates a positive feedback loop between Akt and mTORC2

## Graphical Abstract

**Figure:**
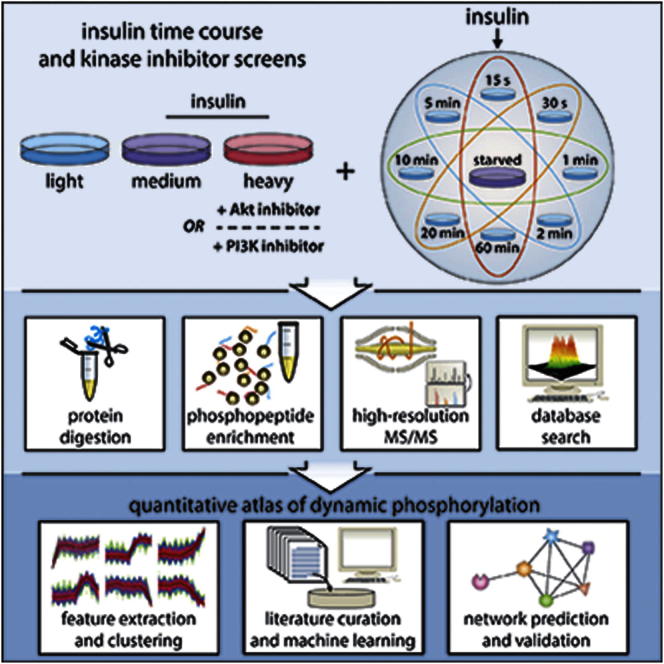

## Introduction

Insulin plays a major role in controlling metabolic homeostasis, cellular growth, proliferation, and survival, and defects in the mechanisms underlying these processes contribute to a range of diseases including type 2 diabetes, cardiovascular disease, and cancer. Protein phosphorylation plays a major role in almost all insulin-regulated processes. As a member of the receptor tyrosine kinase (RTK) family, the insulin receptor engages the canonical phosphoinositide-3 kinase (PI3K)/Akt (Manning and Cantley, 2007) and mammalian target of rapamycin (mTOR) pathways (Zoncu et al., 2011), which together coordinate many of insulin’s actions. These pathways are a major convergence point for many RTKs, and hyperactivation of the PI3K-Akt and mTOR pathways occur in many human tumors (Laplante and Sabatini, 2012; Yuan and Cantley, 2008).

The mTOR-regulated phosphoproteome was recently investigated using quantitative mass spectrometry (MS). By examining the sensitivity to the mTOR inhibitors Torin1, rapamycin, and Ku-0063794, the mTOR network in HEK293 cells and Tsc2^−/−^ mouse embryonic fibroblasts (MEFs) was assessed to a depth of 4,251 and 7,255 phosphosites, respectively (Hsu et al., 2011; Yu et al., 2011). Surprisingly, few studies have examined the diversity of the Akt network on a global scale. It is reported that Akt phosphorylates in excess of 100 substrates; however, many of these sites were identified using targeted approaches. Moreover, the extent to which hormones such as insulin rely on signaling nodes other than the canonical PI3K/Akt pathway for their physiologic functions remains uncertain. Therefore, to obtain a more complete view of the PI3K/Akt network, it would be desirable to take an unbiased approach using quantitative MS. To delineate the complexity of the insulin signaling network, we generated dynamic maps of the insulin-regulated phosphoproteome in insulin-responsive 3T3-L1 adipocytes. To investigate the role played by the key insulin-regulated kinases Akt and PI3K, we combined these approaches with pharmacological inhibitors targeting these signaling nodes. This revealed a complex single-cell phosphorylation network comprising 37,248 phosphorylation sites on 5,705 proteins, 15% of which were insulin regulated. There was considerable diversity in the temporal pattern of phosphorylation among different insulin-responsive targets. We demonstrate the utility of these large phosphoproteome data sets for the identification of insulin-regulated phosphorylation sites by developing and applying a general in silico machine learning approach to systematically predict kinase-substrate relationships. This strategy builds upon existing methods based on identification of kinase consensus motifs (Linding et al., 2008; Obata et al., 2000; Obenauer et al., 2003) or chemical-genetic approaches (Blethrow et al., 2008; Chi et al., 2008) while overcoming problems associated with consensus motif redundancy and preserving the specificity and context of the cellular environment.

## Results

### Quantification of the Adipocyte Phosphoproteome

**Figure 1:**
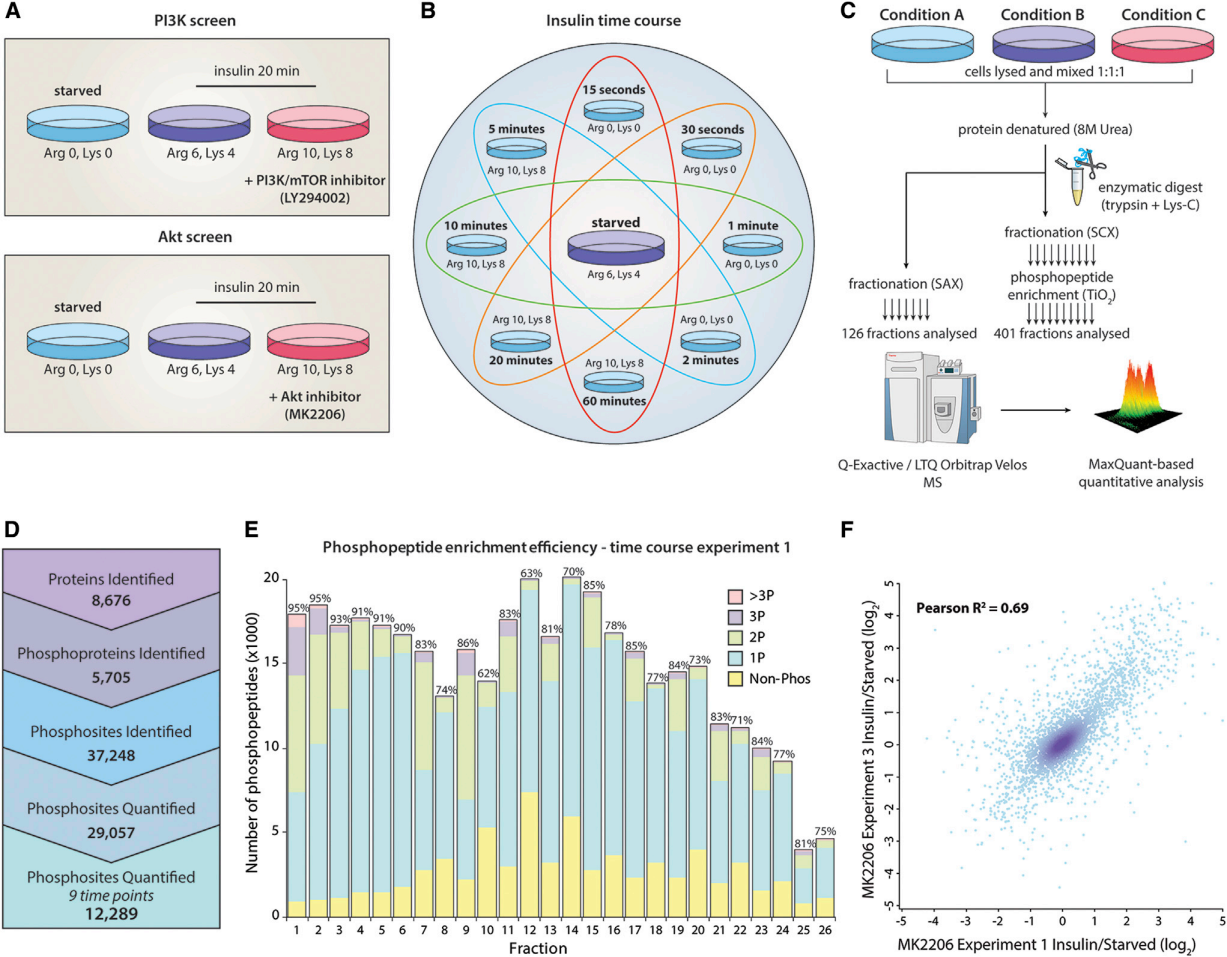
Quantification of the Insulin-Regulated Phosphoproteome using Tandem Mass Spectrometry (A) Experimental design of inhibitor screens. (B) Experimental design of temporal phosphoproteome screen. (C) Workflow for the proteome and phosphoproteome analysis. (D) Summary of the quantified phosphoproteome and proteome. (E) Efficiency of phosphopeptide enrichment for a representative experiment. Above each bar is the percent of the total identified peptides in each fraction that were phosphorylated. (F) Quantitative reproducibility for a representative experiment of one of the large-scale MS studies. See also Figures S1 and S2 and Tables S1, S2, and S3.

To characterize the insulin signaling network we applied two distinct quantitative phosphoproteomics strategies using stable isotope labeling with amino acids in cell culture (SILAC) (Ong and Mann, 2006). In the first set of experiments, we quantified the insulin-regulated phosphoproteome using two potent and selective kinase inhibitors targeting Akt (MK2206) and PI3K/mTOR (LY294002) (Figure 1A). In the second series of experiments we employed a multiplexed SILAC approach to interrogate the temporal profile of insulin signaling over a wide temporal dynamic range spanning 15 s to 60 min (Figure 1B). All MS experiments were performed in triplicate. We achieved high confidence and deep coverage of the phosphoproteome by analyzing a large number of phosphopeptide-enriched fractions and by taking advantage of recent developments in MS hardware (Michalski et al., 2011) and software (Cox and Mann, 2008; Cox et al., 2011) (Figure 1C). In total, 7,441,138 high-resolution (Orbitrap higher-energy collision dissociation [HCD]) spectra were acquired, resulting in the identification of 38,901 unique phosphopeptides corresponding to 37,248 phosphorylation sites on 5,705 proteins (Figure 1D and Tables S1 and S2), placing this among the largest phosphoproteomes reported. Phosphopeptide identification confidence was high, with 95% of all peptides identified having an absolute mass deviation less than 2 ppm (Figures S1A and S1B).

Our phosphopeptide enrichment was highly efficient and selective, since phosphopeptides consisted of an average of 83% of the total peptides identified from the phosphopeptide-enriched samples (Figure 1E) and reproducibility of phosphopeptide quantitation between biological replicates was good (Figure 1F). Single-, double-, triple-, and higher-phosphorylated peptides represented 72%, 23%, 4% and <1% of the total phosphopeptides, respectively (Figure S1C). Based on the peptide-spectral match and the number of potential phosphorylation sites in each peptide, MaxQuant derived a phosphorylation site localization probability score (Olsen et al., 2006). We divided the phosphoproteome into four categories based on this probability score: class I (>0.75), class II (0.75–0.5), class III (0.5–0.25), and class IV (<0.25). Importantly, due to the 1% false discovery rate (FDR) applied at the peptide (peptide-spectral matching), protein (protein group assembly), and site (posttranslational modification [PTM] assignment) levels (Cox and Mann, 2008), the probability that all peptides are phosphorylated is greater than 99%. Using this classification, 63% of the sites in our phosphoproteome were accurately localized (class I, Figure S1D) (median localization probability for all sites was 0.96). Assessment of class I phosphopeptides revealed a distribution of 87.8% phosphoserine, 11.4% phosphothreonine, and 0.8% phosphotyrosine (Figure S1D) residues, which is similar to previously reported phosphorylated amino acid distributions (Olsen et al., 2006).

### A Ranked Abundance Atlas of Adipocyte Protein Expression

**Figure 2:**
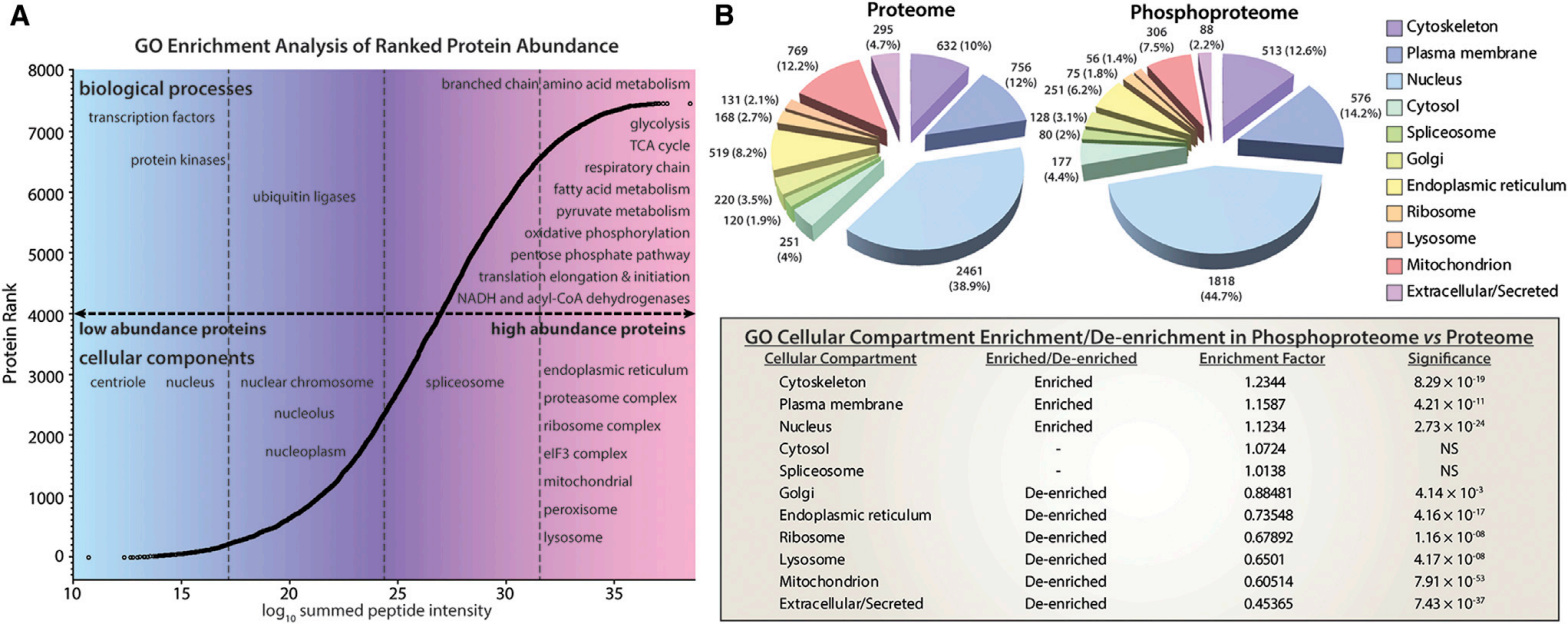
Dynamic Range and Enrichment of GO Terms in Adipocyte Proteome and Phosphoproteome (A) Abundance of detected proteome was estimated using the summed peptide intensities of each protein, and proteins were ranked by abundance and divided into four quartiles. Enrichment of protein GO terms (biological process and cellular component) in each protein abundance quartile was assessed by Fisher’s exact test (FDR < 0.01 following Benjamini-Hochberg correction). The position of GO terms along the horizontal axis represents enrichment of these terms within the respective protein abundance quartile. (B) Frequency and significance of enrichment or de-enrichment of GOCC terms in the proteome and phosphoproteome (Fisher’s exact test, FDR < 0.01 after Benjamini-Hochberg correction). See also Table S4.

In parallel with the phosphoproteome studies, we retained a fraction of the peptides for total proteome analysis by MS following strong anion exchange chromatography (Figure 1C) (Wiśniewski et al., 2009), allowing us to place identified phosphoproteins in the context of the adipocyte proteome. Combined with the phosphoproteome data, we identified a total of 8,676 proteins in 3T3-L1 adipocytes with a median peptide sequence coverage of 23% (Figure 1D). Utilizing the normalized peptide intensities derived from summation of measured peptide-extracted ion chromatograms (intensity-based absolute quantification [iBAQ]) (Schwanhäusser et al., 2011), we quantified and ranked the abundance of the 3T3-L1 adipocyte proteome (Table S4 and Figure 2A). These data revealed that this cell line is highly specialized in metabolic processes and may have preserved many metabolic functions of the tissue of origin. In addition to metabolic enzymes, two fatty acid binding proteins (FABP4 and FABP5) were among the top 10 most abundant proteins in the adipocyte, comparable in expression level to the core nucleosome proteins H4 and H2B (Table S4). Hence, this compendium of ranked protein abundance should serve as a useful resource for cell biologists.

### Comparative Analysis of Proteome and Phosphoproteome in 3T3-L1 Adipocytes

Comparison of the proteome and phosphoproteome revealed an overrepresentation of phosphoproteins associated with the cytoskeleton, plasma membrane (PM), and nucleus, consistent with the importance of phosphorylation as a key functional modulator at these locations (Figure 2B). Enrichment of phosphoproteins in the PM might be anticipated since this compartment is a hub for protein signaling. Surprisingly, such enrichment has not been observed in previous studies, with one study showing an underrepresentation of plasma membrane proteins (Olsen et al., 2006). It was proposed that this occurred due to insufficient phosphoproteome coverage to sample low-abundance membrane proteins. The enrichment of these proteins in our data set reflects the absence of bias against low-abundance PM proteins and a substantial depth of coverage likely achieved. In contrast, there was a relative paucity of phosphoproteins associated with mitochondria and lysosomes. These data suggest that protein kinases likely have connectivity with a broader range of substrates at the PM and nucleus compared with these other locations. Moreover, since the lysosomal surface is a well-known signaling hub for mTOR and extracellular signal-regulated kinase (ERK) (Bar-Peled and Sabatini, 2012), this finding raises the possibility that resident lysosomal proteins are not the principal substrates for these kinases at this location.

To further assess the phosphoproteome coverage of our data, we mapped the proteins found to contain phosphorylation sites onto the ranked proteome abundance (Figure 2A). We observed good coverage of proteins spanning the entire abundance of the measured proteome, with a relative enrichment of low-abundance proteins (p = 8.5 × 10^−8^, Wilcoxon rank-sum test). Of course, phosphorylation is not limited to single sites within a protein, so even with near-exhaustive coverage of phosphorylated proteins, there will likely be many phosphorylation sites that remain out of reach with the current technology or that are only present at measurable stoichiometry following unique cellular stimulus.

### Comparison with In Vivo Mouse Phosphoproteome

We next compared our data set with a study performed in nine mouse tissues (Huttlin et al., 2010). We observed considerable overlap of the identified phosphoproteomes among our study and the mouse tissue study. Our study identified 66% of the total mouse tissue phosphoproteins (Figure S2A) and 90% of the phosphoproteins from brown adipose tissue. Combining the data from these two studies alone identified 57,644 phosphorylation sites on over 7,800 proteins (Figure S2B). Phosphosites identified from brain, spleen, and heart were de-enriched in our study, while those from insulin target tissues (liver, pancreas, and brown adipose tissue) were most enriched. This suggests that cells from these tissues may share a subset of common phosphoprotein machinery and emphasizes the cell-type-specific nature of protein phosphorylation.

We identified several phosphorylation sites known to be important to the actions of insulin and growth factor signaling in general, such as the activating T loop phosphorylation site of Akt (T308) and the hydrophobic motif (HM) of Akt1 (S473) not identified in the mouse tissue phosphoproteome study. The detection of these and other phosphorylation sites in our data highlights the importance of system perturbations using growth factors or other stimuli to activate signaling networks when studying the phosphoproteome. Under resting or starved conditions, the stoichiometry of many growth factor-regulated phosphorylation sites is known to be low, making their detection unlikely if not impossible.

### The PI3K/Akt/mTOR Network

**Figure 3:**
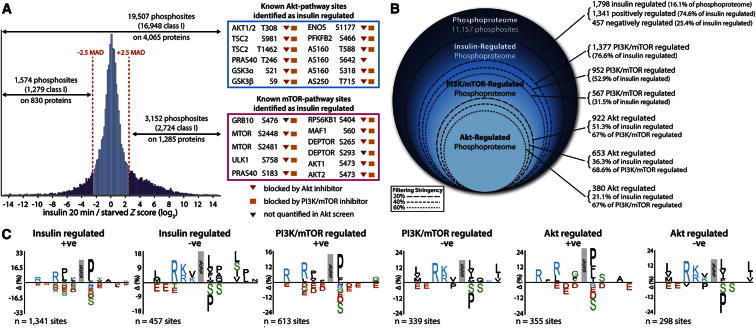
Quantification of Insulin/PI3K/Akt-Sensitive Phosphoproteomes (A) Distribution of all quantified phosphopeptides in inhibitor screens and insulin-responsive sites (purple). Known Akt-mediated (blue box) and mTOR-mediated (pink box) phosphosites regulated by insulin. Phosphosites are annotated as PI3K/mTOR- (orange squares) or Akt (red triangles)-dependent if their insulin-mediated phosphorylation was reversed by >40%. (B) PI3K- and Akt-regulated components of the insulin-regulated phosphoproteome. A subset of these were considered PI3K regulated if they were inhibited by LY294002 by more than the indicated thresholds and Akt regulated if they were also inhibited by MK2206. (C) Sequence logos for phosphorylation sites that were insulin regulated.

In the studies using PI3K and Akt inhibitors, we quantified 19,507 unique phosphorylation sites on 4,065 proteins (FDR < 1%; Figure 3A), and around 15% of the sites quantified in either screen were regulated by insulin (Figure 3A). Many of the insulin-regulated sites belong to the PI3K/Akt/mTOR pathway, and phosphorylation of most of these substrates was inhibited by MK2206 and LY294002, reflecting that these inhibitors target sequential proximal steps in the canonical insulin signaling pathway.

Applying a threshold of 2.5 median absolute deviations to the log_2_-transformed data (roughly equating to a 2-fold change in this data set) revealed 3,152 positively regulated and 1,574 negatively insulin-regulated phosphorylation sites on 1,285 and 830 proteins, respectively (Figure 3A). To compare phosphorylation affected by the PI3K/mTOR and Akt inhibitors, we filtered class I phosphorylation sites that were quantified in at least one biological replicate from each of the screens, and for increased robustness we filtered the data such that phosphorylation sites had to be regulated in the same direction in both inhibitor screens. This revealed that over 50% of the insulin-regulated phosphoproteome was blocked by the PI3K/mTOR inhibitor (Figure 3B and Table S1), confirming the dominance of these pathways in insulin action. We iteratively explored multiple stringencies for PI3K-Akt inhibition and observed that, regardless of the filtering stringency utilized, the Akt inhibitor blocked around 67% of the insulin-regulated, PI3K-sensitive phosphorylation sites, suggesting that the majority of PI3K-mediated growth factor signaling is coordinated through Akt-dependent mechanisms.

### Site-Specific Motifs Enriched in the Insulin-Regulated Phosphoproteome

Enrichment of amino acids surrounding the phosphorylated residues can be useful in revealing the broad classes of kinases that are active in the context of the sample analyzed (Colaert et al., 2009). Among the insulin-stimulated phosphorylation sites, we found a significant (p < 0.01) overrepresentation of proline-directed and basophilic-containing motifs and, correspondingly, fewer acidophilic sequences (Figure 3C). Proline-directed sites are phosphorylated by several kinases, including mTOR, mitogen-activated protein kinases (MAPKs), and cyclin-dependent kinases (CDKs), while basophilic sequences are phosphorylated by the AGC kinases Akt, serum and glucocorticoid-induced kinase (SGK), p70S6K, and p90S6K, among others. Many of these kinases are regulated by growth factors, so enrichment of these motifs in the insulin-regulated phosphoproteome is expected. Acidophilic kinases include casein kinases 1 and 2. The de-enrichment of these sequences in the insulin-regulated phosphoproteome indicates that acidophilic kinases are constitutively active in adipocytes.

The consensus motifs for insulin-regulated, PI3K/mTOR-regulated, and Akt-regulated phosphorylation sites were found to be remarkably similar, with only modest differences between these classes of phosphosites (Figure 3C). For example, Akt-stimulated phosphorylation sites appear to have a greater preference toward threonine and serine residues in the +1 and +2 positions, respectively, when compared with PI3K/mTOR-stimulated sites. This similarity likely reflects the dominance of Akt and associated AGC kinases as downstream components of the PI3K/mTOR pathways and the tight connectivity of these signaling networks.

### Temporal Clustering of the Insulin-Regulated Phosphoproteome

**Figure 4:**
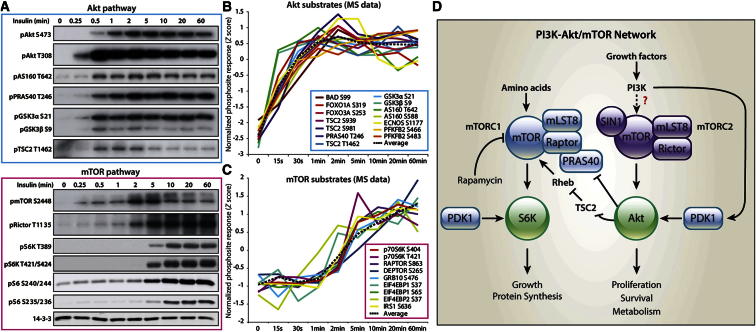
Dynamic Quantitative Analysis of Akt/mTOR Networks (A) Immunoblot analysis of adipocytes following different insulin-stimulated time points for proteins known to belong to the Akt (blue) and mTOR (pink) pathways. (B and C) Temporal profiles generated from SILAC-MS data for known direct Akt (B) and mTOR (C) substrates. (D) Network model depicting the activation of Akt, mTORC1, and mTORC2 by growth factors. See also Figure S3.

We next investigated the dynamics of insulin-regulated phosphorylation (Figure 1B). We initially focused on temporal profiles of phosphorylation sites known to belong to pathways regulated by Akt and mTORC1 since, according to the data from our inhibitor screens, the PI3K-Akt/mTOR axis accounts for over half of the insulin-regulated phosphoproteome. We observed segregation in the temporal profiles of phosphorylation sites belonging to these two pathways (Figure 4A), with Akt substrates phosphorylated rapidly, reaching a maximum within 1 min of insulin stimulation (Figure 4B). In contrast, mTORC1 substrates were phosphorylated substantially slower (Figure 4C). This latency may reflect differences in the molecular mechanisms governing activation of the kinases regulating these pathways (Figure 4D) or spatial differences in kinase and substrate localization.

**Figure 5:**
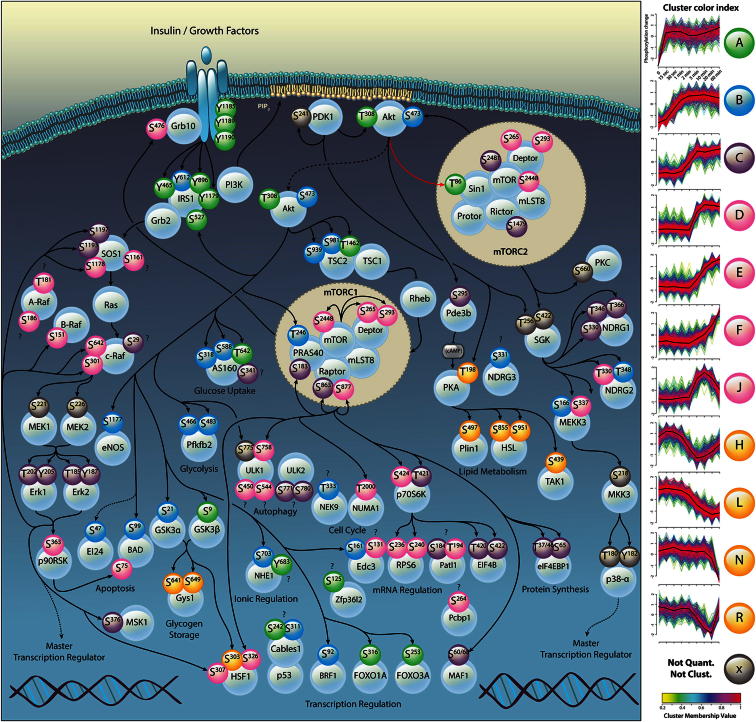
Temporal Phosphorylation in Response to Insulin Reveals Signaling Network Topology Data from the literature were used to construct a cell signaling network. Proteins identified in this study were annotated with their respective insulin-dependent phosphorylation sites color coded according to the temporal patterns derived from unsupervised clustering (fuzzy *c*-means), shown at the right. Complete clusters (A–R) are shown in Figure S3 and listed in Table S2. See also Figures S4 and S5.

To classify the temporal patterns of phosphorylation in an unbiased manner, we performed unsupervised clustering (fuzzy *c-*means) (Futschik and Carlisle, 2005) of the time course data. Approximately 50% of the temporal phosphorylation patterns showed a sustained increase with insulin, while the remainder were either transient or decreased (Figure S3). To investigate the utility of these temporal data for identifying the proximity of signaling components, we constructed a network comprising over 100 well-described phosphorylation sites and overlaid the unsupervised clustering results (Figure 5). Examination of this network reveals functional temporal clusters reflecting signaling connectivity between the nodes. For example, upstream elements such as the tyrosine-phosphorylated insulin receptor and IRS1, as well as Akt regulatory sites, were found in the most rapid cluster (cluster A), while MEK1/MEK2-mediated activation of ERK1 and ERK2 (cluster C) and the ERK substrate p90RSK (cluster J) were slower (Figure 5).

Interestingly, all well-characterized Akt substrates were found in the fastest clusters (A and B), together with the activation sites of Akt (T308 and S473), while p70S6K and its substrate (S6) were found together in slower clusters (clusters D–F) (Figure 5). This suggests that kinase activation alone is the rate-limiting step for substrate phosphorylation and that in vivo substrate phosphorylation kinetics could be used as a powerful predictor for identifying previously unknown, physiological kinase-substrate relationships. This is exemplified by phosphorylation of Bcl2 antagonist of cell death (BAD), since BAD contains both Akt (S99, cluster B) and p90RSK (S75, cluster D) phosphorylation sites (Figure 5). Given that Akt is found in the most rapid cluster, one explanation for this temporal specificity is that Akt-dependent phosphorylation confers conformational changes within substrates, thereby promoting access to other kinases; alternatively, differential subcellular localization of substrates might limit the kinetics of phosphorylation. The rapid phosphorylation of Akt substrates at distal cellular locations (e.g., forkhead box-O [FOXO]), however, argues against this. Another possibility is that this occurs due to carefully tuned, intrinsic-specific activities of each kinase toward different substrates. The deregulation of this finely tuned specificity (due, for example, to hyperactive or upregulated kinase expression, as is often observed in tumors [Yuan and Cantley, 2008; Zoncu et al., 2011]) may therefore explain how dysregulated signaling can lead to uncontrolled tumor growth and proliferation. Hence, temporal information is likely to be a revealing determinant of network connectivity.

### Predicting Kinase Substrates from Integrated Global Phosphoproteomics Data

A major challenge in studying protein phosphorylation is defining physiological kinase-substrate relationships. This is difficult in light of the loss of sequence specificity that occurs for many kinases in vitro. For example, the evolutionarily related AGC kinases Akt and p70S6K phosphorylate substrates with indistinguishable motifs in vitro (Manning and Cantley, 2007), yet our temporal data clearly segregate known Akt and S6K substrates. Due to the substantial resolution of these closely related kinases, we sought to utilize our phosphoproteomics data to develop an integrative method of enhanced kinase-substrate prediction. To achieve this, we applied a machine learning approach utilizing support vector machines (SVMs) (Ben-Hur et al., 2008) and curated positive training sets from the literature (Tables S2 and S3) to sensitively predict cognate kinase-substrate relationships using the combined data from both of our large-scale phosphoproteomics screens (Figure S4A). Regarding selection of positive training sets, substrates for which there were multiple lines of evidence from different sources were given preference. We reasoned that careful selection of true positives for the training sets was critical due to the reduced specificity that many protein kinases may have displayed under in vitro assay conditions. Residual phosphorylation sites from the remaining data set were treated as negative examples (see Supplemental Experimental Procedures).

Kinase-substrate prediction classifiers were trained for Akt, mTORC1, and PKA, since these kinases collectively represent a wide range of the actions of insulin and are implicated in a diverse range of biological processes of broad significance. Features used for classification were selected to describe three diverse characteristics of each substrate: (1) the amino acid sequence surrounding the phosphorylated residue, (2) the response of the phosphosite to the PI3K/mTOR and Akt inhibitors (compared with insulin alone), and (3) the temporal profile of phosphorylation in response to insulin (Figures S3 and S4B). Using this approach, we generated a normalized prediction score for Akt, mTORC1, and PKA for each phosphorylation site that was quantified in the time course data set (Table S2). A delta score was also calculated to determine whether the prediction score for a specific substrate was substantially higher for another kinase. We then used a Pareto ranking approach to objectively incorporate both ensemble prediction scores and delta scores for prioritizing and ranking potential Akt, mTORC1, and PKA substrates for follow-up molecular characterization (Figure S5A and Table S2). The average temporal profiles of the top 50 ranked predictions for each kinase closely resemble the established profiles of known substrates (Figure S5B), and the estimated sensitivity and specificity of the approach (Figure S5C) indicated that the SVMs are successfully predicting substrates based on their temporal characteristics.

### Insulin Regulates Diverse Cellular Processes through Convergence on Common Targets

While the high-ranking Akt, mTORC1, and PKA predictions from our machine learning approach included many known substrates for each kinase, there were also many phosphorylation sites not previously recognized as part of the insulin signaling network. Many of these previously unrealized phosphorylation sites likely represent previously unrealized actions of insulin. For instance, putative mTORC1 sites in Ulk1 and Ulk2, the ubiquitin-specific processing protease 32 (Usp32), the La ribonucleoprotein domain family member 4B (Larp4B), the poly(rC)-binding protein 1 (Pcbp1), and topoisomerase II homolog 1 (Patl1) (Figure S5A and Table S2) suggest possible mechanisms of regulation of autophagy, ubiquitin-dependent protein turnover, and messenger RNA (mRNA) stability. Moreover, we found phosphorylation sites on Patl1 and Larp4B that were predicted to be PKA substrates (Figure S5A). This points to mRNA turnover as a major determinant of insulin-regulated cellular growth and demonstrates that insulin regulates cellular processes by coordinated control of numerous signaling pathways that converge on common targets. Consistent with this, Akt phosphorylation of Enhancer of mRNA-decapping protein 3 (Edc3) and butyrate response factor 1 (Brf1) regulate mRNA stability in mammalian cells (Larance et al., 2010; Schmidlin et al., 2004) (Figures 5 and S5A). Other predicted substrates imply nascent areas of mTOR biology, including vesicle trafficking and endocytosis via the Rab effector Micall1, the Rab guanine nucleotide exchange factor Dennd4C, and the Rab binding protein Plekhm1, as well as uncharacterized sites on a-Raf and c-Raf, which may point to crosstalk between mTOR and MAPK signaling pathways.

Candidate Akt substrates include butyrate response factor 2 (Brf2), an mRNA-binding protein that promotes mRNA deadenylation and degradation. A homologous site on Brf1 is a known Akt substrate (Schmidlin et al., 2004), supporting our prediction that Brf2 is likely an Akt substrate. Other putative Akt substrates are the insulin receptor substrates 1 and 2 (IRS1/IRS2), Stress-activated map kinase-interacting protein 1 (SIN1), and etoposide-induced 2.4 kb transcript (EI24) (Figure S5A). EI24 is a p53-induced DNA damage response gene involved in growth suppression and apoptosis, located on a region in the human genome most consistently deleted in solid tumors (11q23) (Gu et al., 2000). We verified the phosphorylation of EI24 in HEK293 cells and showed that this was blocked by MK2206, but not by rapamycin (data not shown). The potential regulation of EI24 by Akt is of considerable interest, as it may represent a mechanism by which Akt mediates cell survival and apoptosis, an important finding in light of the clear role of Akt in the context of cellular proliferation and cancer.

### Identification of SIN1 as a Direct Akt Substrate that Regulates mTORC2 Activity

**Figure 6:**
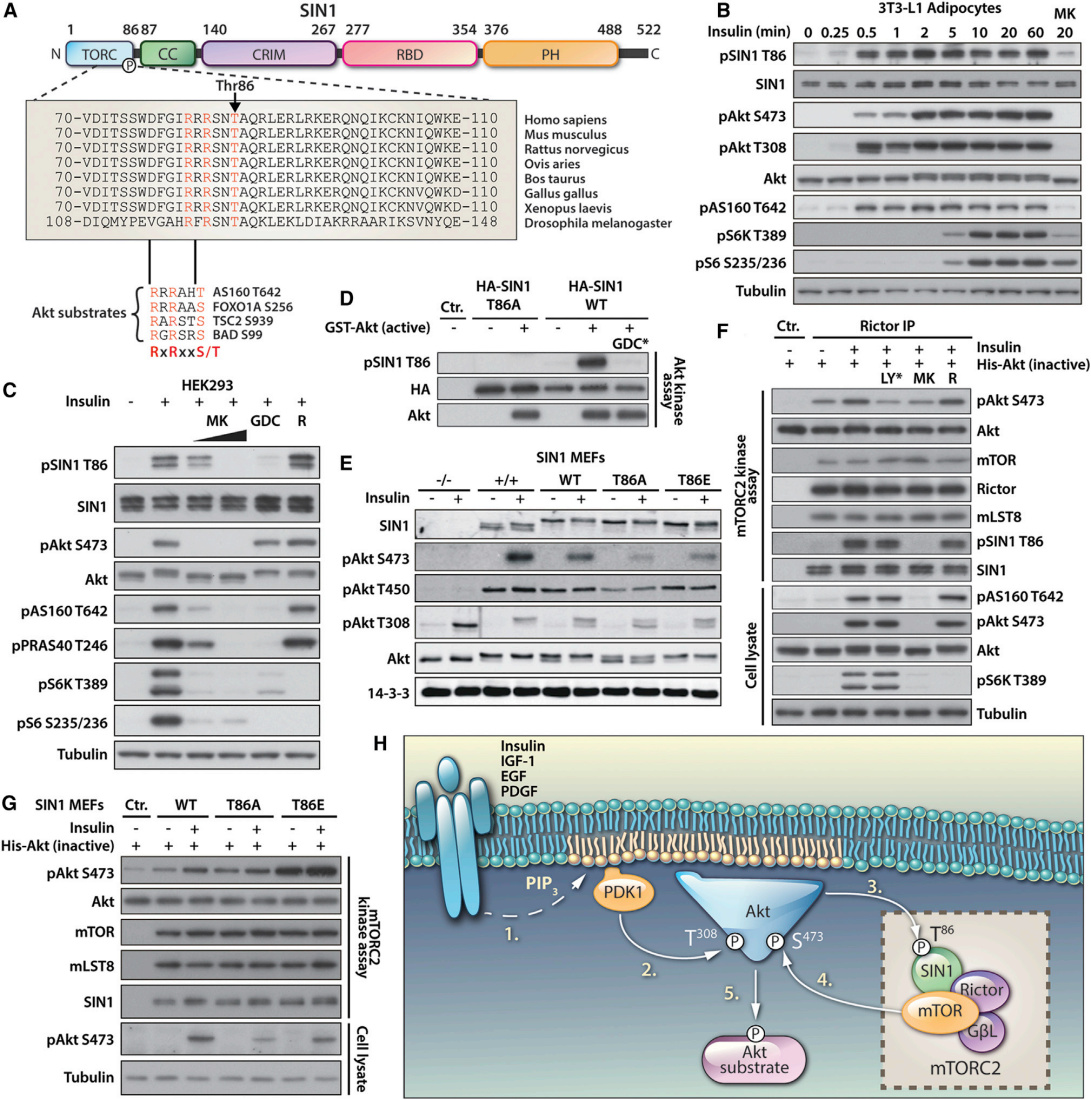
Akt Is the Physiological Kinase for SIN1 T86, and its Phosphorylation Directly Regulates mTORC2 Activity (A) SIN1 domain structure and sequence homology of the region surrounding T86. TORC, putative mTORC-binding domain; CC, coiled-coil domain; CRIM, conserved region in the middle domain; RBD, Raf-like Ras-binding domain; PH, pleckstrin homology domain. Enlarged is the region containing the insulin-responsive phosphorylation site, T86. Residues surrounding several other known Akt substrates (AS160 T642, FOXO1A S256, TSC2 S939 and BAD S99) are shown. (B) Endogenous SIN1 is rapidly phosphorylated in response to insulin and blocked by pretreatment with the Akt inhibitor MK2206. 3T3-L1 adipocytes were serum starved, treated with MK2206 (MK; 10 μM, 30 min), and stimulated with insulin (100 nM) for the indicated times, and samples were assessed by immunoblotting. (C) Insulin-stimulated phosphorylation of endogenous SIN1 T86 is blocked by MK2206 and GDC-0068, but not by rapamycin. HEK293 cells were serum starved overnight, treated with MK2206 (MK; 10 μM and 1 μM), GDC-0068 (GDC;10 μM), or rapamycin (50 nM) followed by insulin (200 nM, 10 min), and samples were analyzed by immunoblotting. (D) Akt in vitro kinase assay performed using recombinant GST-Akt results in specific phosphorylation of SIN1 at T86 and is blocked by GDC-0068 (GDC^*^; 10 μM) added to the in vitro kinase reaction. (E) Expression of SIN1, but not SIN1 T86A mutant, in SIN1^−/−^ MEFs rescues mTORC2-dependent signaling. SIN1 WT or phosphomutants (T86A, T86E) were expressed in SIN1^−/−^ MEFs, and cells were selected by FACS. Cell lines were serum starved and stimulated with insulin, and samples were analyzed by immunoblotting. (F) In vitro kinase activity of endogenous mTORC2 isolated from cells is enhanced by insulin stimulation (200 nM, 10 min) and blocked by pretreatment of cells with MK2206 (MK; 10 μM), but not rapamycin (R; 50 nM). LY294002 (LY^*^; 15 μM) was added directly to the in vitro kinase assay. (G) mTORC2 isolated from SIN1^−/−^ MEFs reconstituted with SIN1 WT or phosphomutants (T86A, T86E) displays differential growth factor-stimulated kinase activity in in vitro kinase assay, with enhanced mTORC2 activity isolated from T86E phosphomimetic mutants. (H) Model depicting growth factor-dependent activation of mTORC2 mediated by Akt phosphorylation of SIN1. See also Figure S6.

One predicted Akt substrate of particular interest was SIN1, an indispensible subunit of mTORC2 (Figure 4D) required for complex assembly and mTORC2 kinase activity (Jacinto et al., 2006). We identified rapid phosphorylation of SIN1 on T86 in response to insulin in our MS-based proteomics experiments (cluster A; Figure 5) along with Akt (T308) and numerous Akt substrates (area under curve [AUC] = 0.86; Figure S4B). SIN1 T86 is located in a highly conserved region of the protein (Figure 6A). We verified the phosphorylation of SIN1 T86 by generating a phosphospecific antibody and confirmed that SIN1 T86 phosphorylation in response to insulin occurred on a timescale similar to that of Akt S473 and Akt substrates AS160 and GSK3 in 3T3-L1 adipocytes and HEK293 cells. However, phosphorylation of SIN1 was temporally distinct from S6K and the S6K substrate S6 (Figures 6B and S6A); it was inhibited dose dependently by the allosteric Akt inhibitor MK2206 and blocked by the ATP-competitive Akt inhibitor GDC-0068, but not by the mTORC1 inhibitor rapamycin (Figures 6B and 6C). Moreover, Akt phosphorylated SIN1 T86 in vitro, and this phosphorylation was blocked by GDC-0068 (Figure 6D). Together, these findings confirm that Akt is the physiological kinase of SIN1 T86.

We predicted that Akt-mediated phosphorylation of SIN1 might be involved in regulating the activity of mTORC2 because its phosphorylation occurred on a timescale that would support such a function relative to Akt S473 phosphorylation, and PI3K inhibition impairs the activation of mTORC2 (Frias et al., 2006). One way in which SIN1 T86 might regulate mTORC2 is by affecting formation of the complex, since SIN1 is essential for mTORC2 complex stability (Jacinto et al., 2006). To test this, we expressed wild-type (WT) or phosphomutant (T86A) SIN1 in SIN1^−/−^ MEFs and selected for cells expressing SIN1 similar to endogenous levels. We found that SIN1 T86A was equally capable of forming mTORC2, since immunoprecipitation of Rictor copurified both the phosphomutant and WT SIN1 equally, suggesting that formation and stability of the complex was not impaired (Figure S6B). In contrast, insulin-dependent phosphorylation of Akt S473 was blunted in SIN1 T86A cells, but not in SIN1 WT cells (Figure 6E), suggesting that phosphorylation of SIN1 T86 by Akt directly regulates mTORC2 activity. Consistent with this, substitution of the phosphorylated residue with a phosphomimetic (T86 to glutamic acid, T86E) restored growth factor-dependent mTORC2 signaling (Figures 6E and S6C).

In addition to S473, Akt is also phosphorylated at an additional site (T450) by mTORC2. Consistent with this, Akt T450 phosphorylation was abolished in SIN1^−/−^ cells (Facchinetti et al., 2008) (Figure 6E). Intriguingly, while SIN1 WT and SIN1 T86E completely rescued Akt T450 phosphorylation, this was not the case for SIN1 T86A; however, this diminution was not as marked as that observed for Akt S473 phosphorylation. Collectively, this suggests that mTORC2 has differential substrate sensitivities for T450 versus S473 of Akt in vivo, and phosphorylation of SIN1 on T86 potentiates mTORC2 activity toward these sites. Consequently, ablation of SIN1 phosphorylation at T86 has a greater effect on S473 phosphorylation and, in turn, Akt activation.

We next tested whether Akt directly regulates mTORC2 activity in response to growth factors via the phosphorylation of SIN1 T86, using an mTORC2 in vitro kinase assay with exogenous (inactive) Akt as the substrate. mTORC2 complexes obtained from cells pretreated with the Akt inhibitor MK2206, but not rapamycin, had impaired mTORC2 activity and a concomitant block of SIN1 T86 phosphorylation (Figure 6F). In this assay, we used the mTOR inhibitor LY294002 as a control by adding the compound directly to the in vitro kinase reaction to block mTORC2 activity.

We further explored whether the phosphorylation of SIN1 T86 alone was sufficient to directly affect the growth factor-enhanced activity of mTORC2 by isolating the complex from the SIN1^−/−^ MEFs rescued with SIN1 WT, T86A, and T86E mutants and performing in vitro kinase assays. mTORC2 isolated from cells containing SIN1 T86A displayed an impaired growth factor increase in kinase activity (Figure 6G). Moreover, overexpression of the phosphomimetic mutant SIN1 T86E resulted in enhanced, and growth factor-independent, mTORC2 kinase activity (Figure 6G). These data indicate that phosphorylation of SIN1 T86 results in a substantial and direct activation of mTORC2 kinase activity.

We propose the following model for the activation of mTORC2 (Figure 6H): (1) growth factors activate PI3K, causing accumulation of PIP_3_ at the PM; (2) Akt translocates to the PM where its activity is increased by phosphorylation at T308 by PDK1; (3) mTORC2, probably via the pleckstrin homology (PH) domain in SIN1 (Pan and Matsuura, 2012; Schroder et al., 2007), also translocates to the PM, and SIN1 is phosphorylated on T86 by Akt, resulting in activation of the complex; (4) active mTORC2 phosphorylates Akt on S473, enhancing and stabilizing the activity of the kinase; and (5) activated Akt continues to phosphorylate other substrates.

## Discussion

Here we provide a quantitative atlas of dynamic protein phosphorylation and show that these large-scale phosphoproteomics data can be integrated using in silico approaches to delineate key topological features of this essential signaling network. These data serve as a resource for future hypothesis-driven research into both known and currently poorly defined actions of insulin and other growth factors that converge upon signaling nodes like Akt that are central to insulin action.

While dysregulated signaling networks downstream of RTKs are a key feature of diabetes, cardiovascular disease, and many types of cancer, there is a paucity of systems-wide quantitative data delineating signaling networks in healthy cells. Hence, the data described herein brings us a step closer to a more complete understanding of disease phenotypes. It is apparent, for example, that the scale of growth factor-regulated protein phosphorylation, in terms of both functional and temporal distribution, is much greater than previously appreciated. This underscores the importance of classifying phosphorylation data using context-specific systems. Therefore, the current study, which focuses on insulin signaling in the adipocyte, will provide an ideal framework with which to move toward more complex signaling networks in tissues comprising multiple different cell types and multiple intercellular communication networks.

Our data highlight the temporal dimension in protein phosphorylation as a key indicator of functional behavior and its modification by extrinsic cues such as growth factors, including insulin. Two major nodes regulated by insulin are mTOR and Akt, each of which controls distinct biological endpoints such as nutrient sensing, protein synthesis, metabolism, and cell survival. In our quantitative phosphoproteome, we observed substantial temporal resolution in the activation of these two pathways. The activation of Akt involves at least eight molecular events consisting of changes in protein localization, protein-protein interaction, and conformation. Despite this, Akt was fully active within 30 s of insulin stimulation, while the activation of mTORC1 was markedly delayed. The resolution of Akt and mTORC1 activity (Figures 4B and 4C) is in contrast to the temporal clustering of Akt and upstream elements (e.g., IR and IRS). While it might be predicted that mTORC1 activation occurs after Akt since mTORC1 is downstream of Akt, the extent of the observed temporal latency is not intuitively obvious from the biochemistry of mTORC1 activation (Laplante and Sabatini, 2012). Moreover, recent studies have demonstrated that, in adipocytes, mTORC1 is activated at the same location in the cell as Akt (Bridges et al., 2012), making it unlikely that this temporal distinction arises from spatial segregation.

To illustrate the utility of these data for the identification of kinase-substrate relationships, we employed a machine learning approach utilizing SVMs. We demonstrated that one of the predicted substrates, SIN1, is a physiological target of Akt. Moreover, we found that the site-specific phosphorylation of SIN1 T86 is responsible for the direct activation of mTORC2 that occurs in response to growth factors. This is intriguing since mTORC2 is typically considered to be upstream of Akt, as it phosphorylates Akt at the hydrophobic motif (Sarbassov et al., 2005), thereby increasing the activity of the kinase (Alessi et al., 1996). Therefore, our data provide mechanistic insight into the acute activation mechanisms of mTORC2 and the architecture of the Akt-mTORC2 signaling network. Previously it was known that, in response to growth factors, Akt translocates to the PM and is activated by PDK1 phosphorylation of Akt at T308 and mTORC2 phosphorylation of Akt at S473 (Figure 4D). Our data reveal that Akt is intimately involved in a positive feedback loop with mTORC2. We demonstrate that by phosphorylating mTORC2 on SIN1 T86, Akt enhances the activity of mTORC2, which in turn results in increased feedback phosphorylation onto Akt (Figure 6H). This model unifies the growth factor-dependent activation of Akt with that of mTORC2 through an Akt-dependent positive feedback process, providing a more complete picture of the activation mechanisms of both mTORC2 and Akt.

Here we have highlighted only a subset of the biological processes that are encapsulated in our data. A cursory analysis reveals numerous other important pathways that are likely to be regulated by insulin, as well as other RTKs that invoke the PI3K/Akt-mTOR pathways. Hence, we expect that many additional cellular targets of insulin will emanate from studies of this kind.

## Experimental Procedures

### Cell Culture and Peptide Preparation

3T3-L1 fibroblasts were triple SILAC labeled (Ong and Mann, 2006), differentiated into adipocytes, and used on days 10–12 of differentiation. All large-scale MS experiments were performed in three biological replicates. For the inhibitor screens, adipocytes were serum starved then treated with 10 μM MK2206, 50 μM LY294002, or vehicle (DMSO) for 30 min followed by 100 nM insulin or vehicle for 20 min at 37°C (Figure 1A). For time course experiments, adipocytes were serum starved then stimulated with vehicle (PBS) or 100 nM insulin for 15 s, 30 s, 1 min, 2 min, 5 min, 10 min, 20 min, or 60 min, and pooling of SILAC-labeled cells resulted in four groups of cells (Figure 1B). Following mixing, proteins were acetone precipitated, resuspended in urea, reduced, alkylated, and digested with endoproteinase Lys-C followed by trypsin. Peptides were desalted and fractionated by strong anion exchange (SAX) (Wiśniewski et al., 2009) for total-proteome analysis or by strong cation exchange (SCX) and TiO_2_ for phosphopeptide analysis (Larsen et al., 2005; Olsen et al., 2006). Eluted peptides were dried under vacuum before being loaded onto in-house packed C_18_ stop and go extraction (STAGE) tips.

### MS

Eluted peptides were resuspended in MS loading buffer (2% MeCN, 0.3% trifluoroacetic acid [TFA]) and loaded onto a 20 cm column with a 75 μM inner diameter, packed in house with 3 μM C_18_ ReproSil particles (Dr. Maisch GmbH). An EASY-nLC system was connected to the mass spectrometer with a nanospray ion source, and peptides were separated with a binary buffer system of 0.5% acetic acid (buffer A) and 80% MeCN plus 0.5% acetic acid (buffer B), at a flow rate of 250 nL/min. Peptides were analyzed on an Orbitrap Velos or Q Exactive benchtop Orbitrap mass spectrometer (Thermo Fisher Scientific). Up to 10 peptides on the Orbitrap Velos or 15 on the Q Exactive were fragmented in the HCD cell and analyzed with high resolution (7,500 at 400 m/z) in the Orbitrap detector. Dynamic exclusion and lock-mass were enabled (m/z 445.120025).

### MS Data Analysis

Raw mass spectrometry data were processed using the MaxQuant software (Cox and Mann, 2008) version 1.2.3.3, using the default settings with minor changes (see Supplemental Experimental Procedures). Database searching was performed using the Andromeda search engine integrated into the MaxQuant environment (Cox et al., 2011) against the mouse international protein index (IPI) database v3.68, concatenated with known contaminants as well as the reversed sequences of all entries. Protein, peptide, and site FDRs were controlled at a maximum of 1%.

### Statistical Analysis

Data analyses were performed using Microsoft Office Excel, the R software environment, and the bioinformatics platform Perseus (Max Planck Institute of Biochemistry, Munich). Gene ontology (GO) annotation enrichment analysis was performed in Perseus, and significance was assessed using Fisher’s exact test. The whole quantified phosphoprotein data set was used as a background data set, and the Benjamini-Hochberg FDR method was used for multiple hypotheses testing (FDR < 0.02). Motif analysis was performed using iceLogo (Colaert et al., 2009), using percent difference for scoring, a significance threshold of 0.01, and a sequence window of 13 amino acids surrounding the phosphorylated residues of class I insulin-regulated phosphorylation sites (Table S1). The non-insulin-regulated phosphoproteome was used as a reference data set.

### Kinase Substrate Prediction using Machine Learning

The workflow of the analysis is shown in Figure S4A. An ensemble of SVMs were trained using positive training sets curated from the literature to recognize Akt, mTORC1, and PKA substrates, using features extracted from the combined analysis of our large-scale phosphoproteomics studies. The fold ratios for each phosphorylation site over the 9 time points were scaled between 0 and 1. The area under the curve (AUC) for each phosphorylation site was calculated (Figure S4B) and subjected to polynomial curve fitting (order = 2). These were used as descriptive features for SVM training. Other features used for SVM training were the average fold ratios for each site across all time points, the fold ratios with insulin in the presence or absence of inhibitors, and the position-specific scoring matrix of amino acids surrounding the phosphorylation site (sequence window of 13 amino acids).

## Supplemental Information

Document S1. Figures S1–S6 and Supplemental Experimental Procedures

Table S1. Inhibitor Screen Insulin-Regulated Phosphorylation Sites, Related to Figure 1 Phosphorylation sites identified as insulin regulated in both the Akt and PI3K screens.

Table S2. All Identified Phosphorylation Sites, Related to Figure 1 All phosphorylation sites identified from combined inhibitor and time course studies.

Table S3. Class I Phosphorylation Sites, Related to Figure 1 Class I phosphorylation sites identified from combined inhibitor and time course studies.

Table S4. Ranked 3T3-L1 Proteome Abundance, Related to Figure 2 Ranked abundance of all proteins identified from the 3T3-L1 adipocyte proteome.
