# Supplementary material for: Dynamic Adipocyte Phosphoproteome Reveals that Akt Directly Regulates mTORC2
Source: Cell Metab. 2013 Jun 4;17(6):1009–20. doi: 10.1016/j.cmet.2013.04.010 (PMC3690479; doi:10.1016/j.cmet.2013.04.010)
Supplement: Document S1. Figures S1–S6 and Supplemental Experimental Procedures [file mmc1.pdf]

Cell Metabolism, Volume *17*

Supplemental Information

Dynamic Adipocyte Phosphoproteome Reveals

that Akt Directly Regulates mTORC2

Sean J. Humphrey, Guang Yang, Pengyi Yang, Daniel J. Fazakerley, Jacqueline Stöckli, Jean Y. Yang, and David E. James

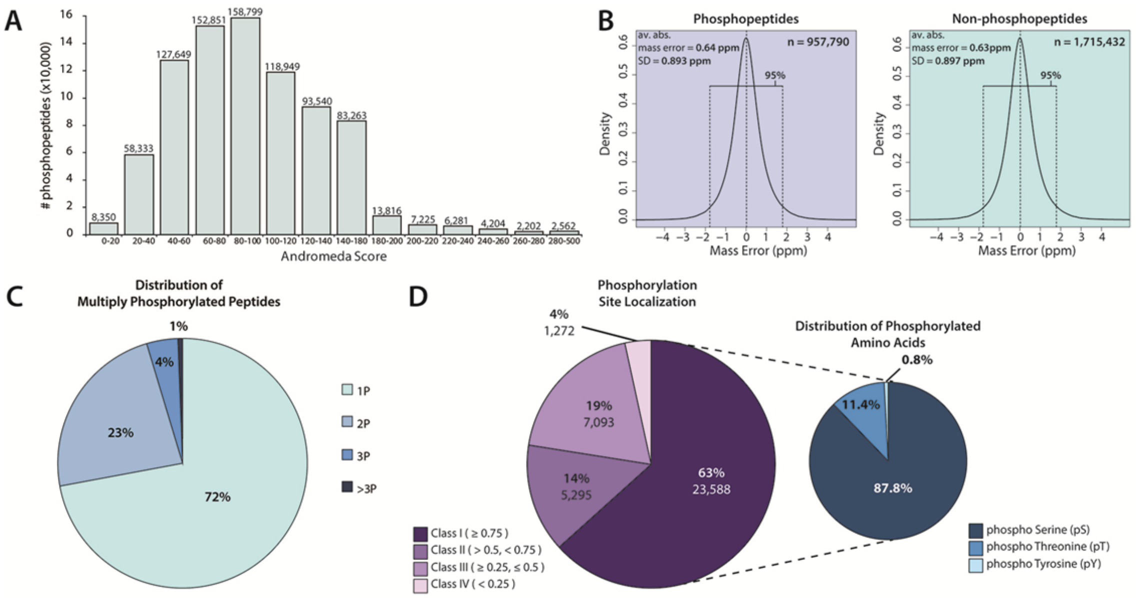

Figure S1. Large-Scale Mass Spectrometry-Based Quantification of the Insulin-Regulated Phosphoproteome, Related to Figure 1

(**A**) Distribution of database search engine (Andromeda) scores for phosphopeptides sequenced and (**B**) measured mass error for all sequenced phosphopeptides and non-phosphopeptides. (**C**) Overall distribution of singly (1P), doubly (2P), triply (3P) or higher phosphopeptides identified in all experiments. (**D**) Distribution of phosphorylation-site localization probabilities for all phosphorylation sites detected in this study (37,248 sites), and the proportion of Serine, Threonine and Tyrosine amino acids phosphorylated in phosphopeptides with high site-localization confidence (Class I phosphorylation sites).

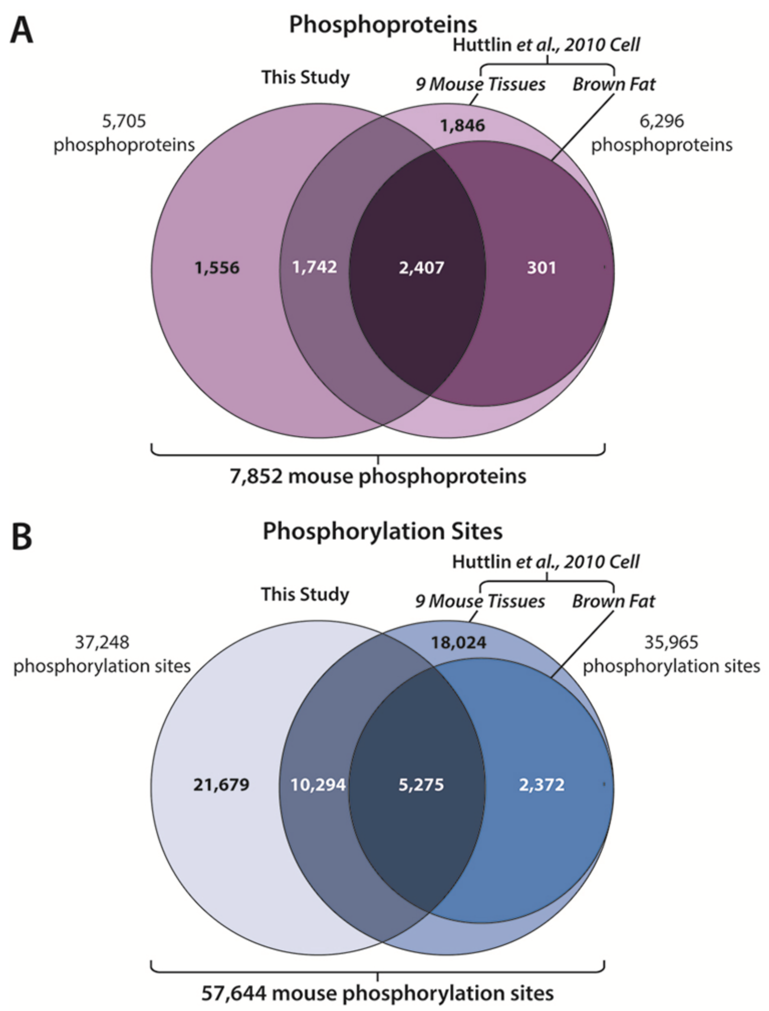

Figure S2. Comparison of Phosphorylation Data Sets from this Study and a Large-Scale Mouse-Tissue Phosphoproteome Study, Related to Figure 1

(**A**) Overlap of phosphoproteins between our dataset and a large mouse tissue phosphoproteome dataset (Huttlin et al., 2010) comparing all 9 tissues, or only brown fat tissue. (**B**) Overlap of phosphorylation sites between the two phosphoproteome datasets, determined using a sequence window of 13 amino acids surrounding phosphorylated residue.

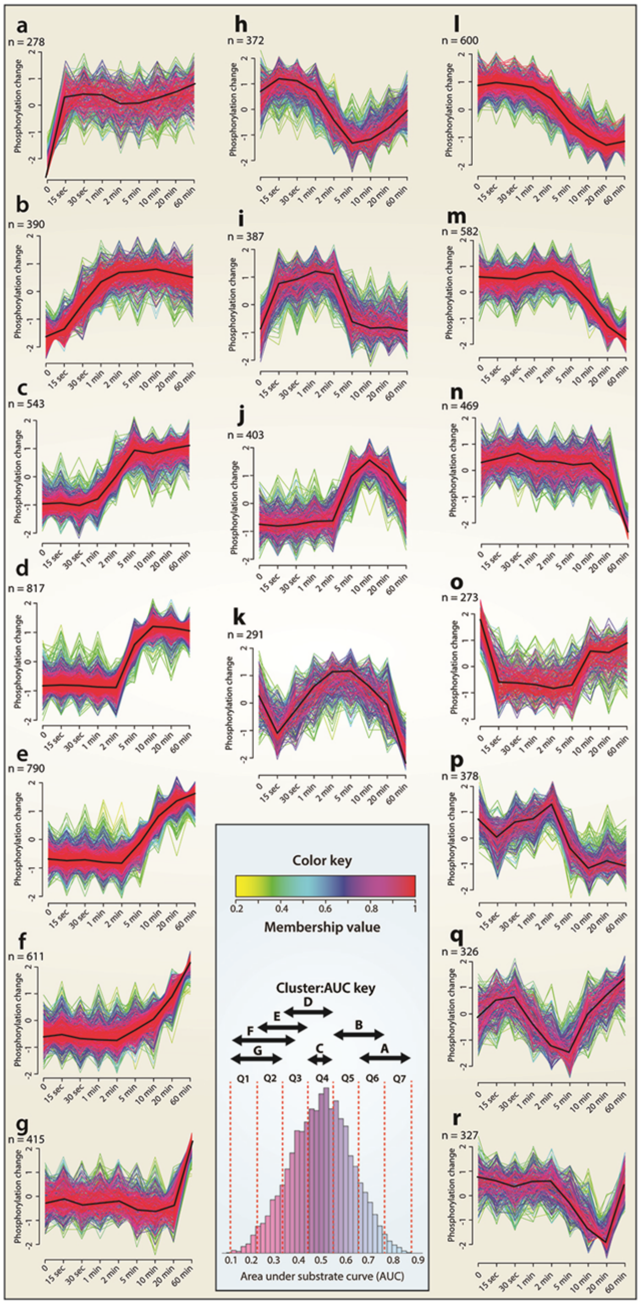

Figure S3. Unsupervised Clustering of Dynamic Phosphorylation Profiles in Response to Insulin, Related to Figure 4

Temporal data spanning 9 time-points was normalized (mean = 0, SD = 1) and assigned to 18 clusters using fuzzy c-means clustering. The number of phosphorylation sites in each cluster is indicated below each cluster letter. The number of phosphorylation sites in each cluster is indicated below each cluster letter. Temporal profiles were coloured by its membership score to the cluster (see color-membership key). The relationship between positively regulated clusters a-g and the Area Under the Curve (AUC) for each cluster was assessed using Fisher exact test (FDR < 0.01 after Benjamini-Hochberg correction).

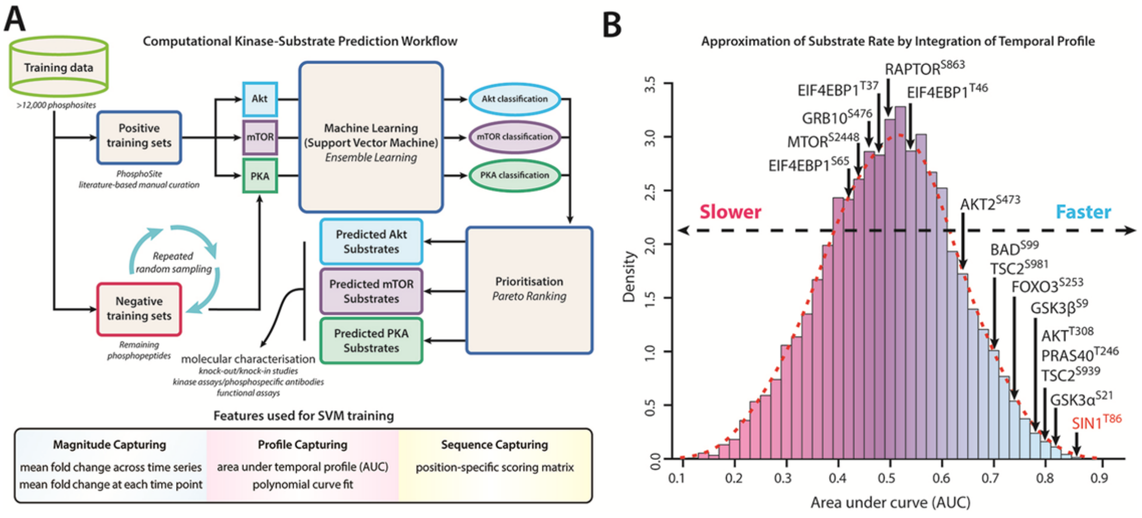

Figure S4. In Silico Methods for Classification of Phosphoproteomics Data and Prediction of Kinase Substrates, Related to Figure 5

(**A**) Flowchart depicting the computational prediction of kinase substrates using integrated phosphoproteomics data sets. The MK2206 and LY294002 screens as well as the large-scale time-course phosphoproteome were used as the training data. An ensemble of Support Vector Machines (SVMs) were trained using a selected group of known substrates for Akt, mTOR and PKA as positive training sets, while negative training sets were created by repeated random sampling from the training data. SVMs were then used to classify the entire phosphoproteome dataset, and predicted substrates were ranked by their substrate prediction scores and delta-scores. Features used for SVM training were designed to capture three properties of substrate phosphorylation including magnitude, temporal profile, and primary amino-acid sequence. (**B**) The area under the curve (AUC) was calculated for each temporal profile and used as a feature for machine learning approach. AUC is a useful descriptive feature for substrate rates. Several substrates belonging to the Akt- (AUC 0.60-0.9) and mTOR-pathways (AUC 0.4-0.6) are indicated.

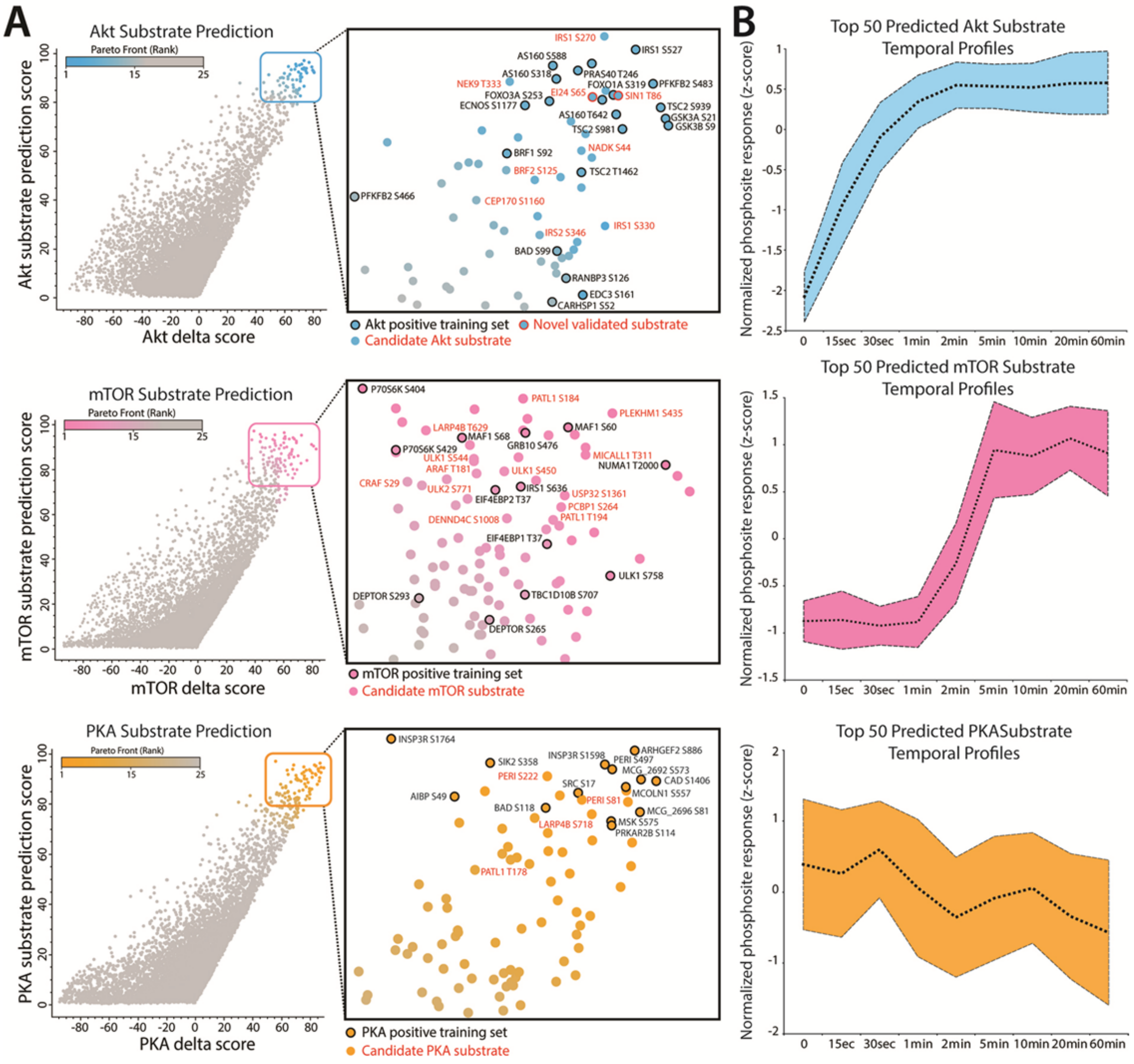

C

10-fold cross-validation of ensemble kinase substrate prediction

SVM features utilized	Sensitivity (Akt)	Sensitivity (mTOR)	Specificity (Akt)	Specificity (mTOR)
Motif	1.00 ± 0.00	0.850 ± 0.254	0.960 ± 0.003	0.827 ± 0.025
Inhibitor phosphoproteome	0.917 ± 0.180	0.800 ± 0.269	0.871 ± 0.010	0.832 ± 0.019
Temporal phosphoproteome	0.976 ± 0.105	0.750± 0.362	0.928 ± 0.013	0.798 ± 0.020
Motif + Inhibitor phosphoproteome	1.00 ± 0.00	0.850 ± 0.254	0.966 ± 0.006	0.903 ± 0.011
Motif + Temporal phosphoproteome	1.00 ± 0.00	0.917 ± 0.180	0.974 ± 0.005	0.876 ± 0.014
Inhibitor + Temporal phosphoproteome	0.976 ± 0.105	0.800 ± 0.219	0.942 ± 0.009	0.842 ± 0.014
All	1.00 ± 0.00	0.883 ± 0.193	0.978 ± 0.003	0.911 ± 0.010

Figure S5. Prioritization of Candidate Kinase Substrates Predicted by Machine Learning, Related to Figure 5

(**A**) Phosphorylation sites quantified in the time-course were ranked by their substrate prediction scores and delta scores for Akt (Blue), mTOR (Pink) and PKA (Orange). The sites with the highest scores were ranked using Pareto fronts. (**B**) Normalized temporal profiles of the top 50 predicted for Akt, mTOR and PKA substrates. Shaded area indicates SD and the dashed line is the average.

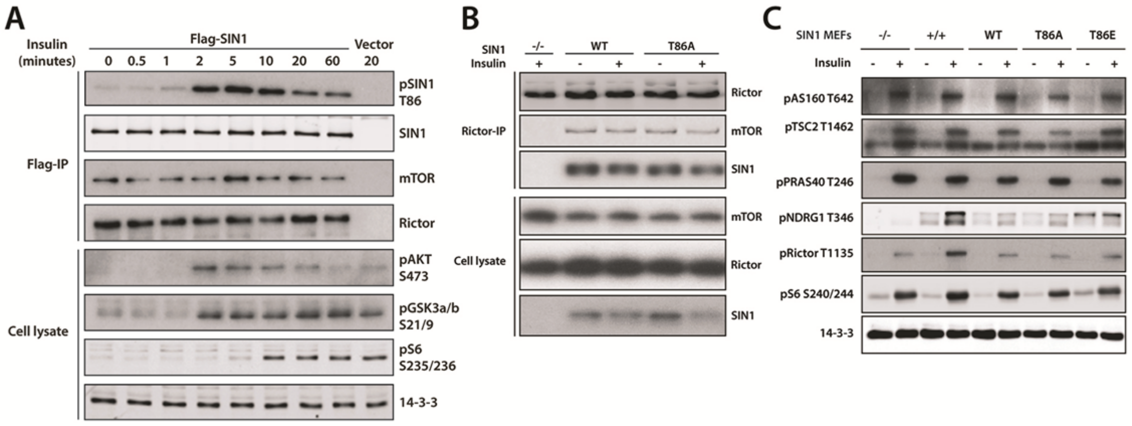

Figure S6. Identification of SIN1 as a Direct Akt Substrate, Related to Figure 6

(**A**) Time-course of SIN1 Thr86 phosphorylation in insulin-stimulated HEK-293T cells. Cells transiently expressing Flag-SIN1 or empty vector were serum starved for 2 h followed by stimulation with insulin (100 nM) for the indicated durations. Flag-SIN1 was immunoprecipitated from cell lysates using the Flag antibody. Immunoprecipitated proteins and total cell lysates were analyzed by immunoblotting using the indicated antibodies. (**B**) mTORC2 complex formation is not affected by SIN1 Thr86. mTORC2 was immunoprecipitated from SIN1 -/- MEF cells stably expressing SIN1wild type (WT) or SIN1 phospho-mutant (T86A) and samples were analysed by immunoblotting for components of mTORC2 complex. (**C**) Signalling in SIN1 -/- MEF cells rescued with SIN1 phospho-mutants. Expression of SIN1but not SIN1 T86A mutant in SIN1 null MEFs rescues mTORC2-dependent signalling. SIN1 wild type (WT) or phospho-mutants (T86A, T86E) were expressed in SIN1 -/- MEFs, and cells selected by FACS as described in *Materials and Methods*. Cell lines were serum starved for 2 h, stimulated with insulin (100 nM, 10 min) and samples analysed by immunoblotting.

Supplemental Experimental Procedures

Cell Culture and Peptide Preparation

3T3-L1 fibroblasts were passaged for six doublings in SILAC DMEM containing three different isotopic versions of arginine and lysine, supplemented with 10% dialyzed FCS, generating 'triple-labelled' SILAC cells as described (Ong and Mann, 2006). SILAC labelled cells were differentiated into adipocytes and used on day 10-12 of differentiation. All large-scale MS experiments were performed in three biological replicates with label switching. For the inhibitor screens, adipocytes were serum-starved, then treated with either 10 µM MK2206, 50 µM LY294002, or vehicle (DMSO) for 30 min, followed by 100 nM insulin or vehicle for 20 min at 37°C (Figure 1A). For time-course experiments, adipocytes were serum starved then stimulated with vehicle (PBS) or 100 nM insulin for 15 sec, 30 sec, 1 min, 2 min, 5 min, 10 min, 20 min, or 60 min, and pooling of SILAC-labelled cells resulting in four groups of cells (Figure 1B). Unstimulated ("starved") cells were present in each group to act as an internal SILAC standard, allowing the generation of temporal profiles. Following mixing, proteins were acetone precipitated, resuspended in urea, reduced, alkylated, and digested with endoproteinase Lys-C followed by trypsin. Peptides were desalted using SepPak tC18 cartridges, and fractionated by Strong Anion Exchange (SAX) for total-proteome analysis or Strong Cation Exchange (SCX) and TiO_2 for phosphopeptide analysis.

Peptide Fractionation and Phosphopeptide Enrichment

For the total proteome analysis, peptides were fractionated by SAX in pipette tip format, as previously described (Wisniewski et al., 2009). For the phosphoproteome analysis, peptides were fractionated by SCX chromatography followed by TiO_2 enrichment (Larsen et al., 2005; Olsen et al., 2006). Briefly, peptides were resuspended in 2 mL 30% MeCN / 0.1% TFA and injected onto an Akta Purifier with a 1 mL Resource S column (GE Healthcare) and fractionated with a linear gradient of 100% buffer A (5 mM KCl, 30% MeCN, 5 mM KH_2PO_4, 0.1% TFA) to 30% buffer B (400 mM KCl, 30% MeCN, 5 mM KH_2PO_4, 0.1% TFA) in 30 min. Fractions including the flow-through were collected and pooled based on absorbance at 215 and 280 nm. Phosphopeptide enrichment was performed as described (Larsen et al., 2005; Olsen et al., 2006). Briefly, Titansphere material (GL Sciences) was suspended in 80% MeCN / 1% TFA containing 30 mg/mL 2,5-dihydroxybenzoic acid, and 3-4 mg was added directly to the SCX fractions. Beads were incubated for 30 min at room temperature with rotation, and collected by centrifugation (5,000 x *g*, 1 min). Titanium dioxide beads were washed three times with 150 µL 60% MeCN / 1% TFA, and transferred to a 200 µL pipette tip containing a plug of C8 (Empore) material. Phosphopeptides were eluted from the beads with NH_4OH / 40% MeCN and dried to near completeness under vacuum before being loaded onto in-house packed C_{18} STAGE tips.

Mass Spectrometry

Eluted peptides were resuspended in MS loading buffer (2% MeCN, 0.3% TFA) and loaded onto a 20 cm column with 75 µM inner diameter, packed in-house with 3 µM C_{18} ReproSil particles (Dr Maisch GmbH). An Easy-nLC system was connected to the mass spectrometer using an 1.9-2.3 kV nano-spray ion source, and peptides were separated with a binary buffer system of 0.5% acetic acid (buffer A) and 80% MeCN / 0.5% acetic acid (buffer B) using linear gradients of buffer B from 5% to 35% over 130 min for phosphoproteome or 240 min for total proteome analysis, at a flow rate of 250 nL/min. Peptides were analysed on an Orbitrap Velos or Q-Exactive benchtop Orbitrap mass spectrometers (Thermo Fisher Scientific). Up to 10 peptides on the Orbitrap Velos or 15 on the Q-Exactive were selected with an isolation window of 2 Th, fragmented in the HCD cell and analysed with high resolution (7500 at 400 m/z) in the Orbitrap detector. Dynamic exclusion was enabled with a duration of 60 s and a mass window of ±7ppm. Lock-mass was enabled using 445.120025.

Data Analysis

Raw mass spectrometry data were processed using the MaxQuant software (Cox and Mann, 2008) version 1.2.3.3 using the default settings with minor changes: Oxidised Methionine (M), Acetylation (Protein N-term) and Phospho (STY) were selected as variable modifications, and Carbamidomethyl (C) as fixed modification, as well as triple SILAC labels (Arg 0/Lys 0, Arg 6/Lys 4, and Arg 10/Lys 8). A maximum of two missed cleavages was permitted, 10 peaks per 100 Da, MS/MS tolerance of 20 ppm, and a minimum peptide length of 6. The "matching between runs" algorithm was enabled with a time window of 2 min to transfer identifications between adjacent fractions, only for samples analysed using the same nanospray conditions. Database searching was performed using the Andromeda search engine integrated into the MaxQuant environment (Cox et al., 2011) against the mouse IPI database v3.68, concatenated with known contaminants and reversed sequences of all entries. Protein, peptide and site FDR thresholds in MaxQuant were each set to a maximum of 1%. Relative protein abundances were estimated using the "intensity-based absolute quantification" (iBAQ) algorithm (Schwanhausser et al., 2011) integrated into the MaxQuant environment. Briefly, protein intensities are derived as the sum of all identified peptide intensities (maximum detector peak intensity of the peptide elution profile including all isotope peaks). Protein intensities are then divided by the number of theoretically observable peptides (fully tryptic peptides, 6-30 amino acids long). The resulting "iBAQ" intensities were log-transformed and used to rank the abundance of detected proteins (Table S3).

Prediction of Kinase Substrates

The workflow of the analysis is shown in Figure S4. An ensemble of SVMs (Ben-Hur et al., 2008) were trained using positive training sets curated from the literature to recognise Akt, mTOR and PKA substrates,

based on features extracted from the combined analysis of our large-scale phosphoproteomics studies. For positive training of these kinase-substrate prediction classifiers, 22, 28 and 17 substrates curated from the literature were used for the kinases respectively. Negative training sets were created by iteratively sampling balanced training sets from the residual data. Because the number of positive training examples is far smaller than the number of negative examples, the class distribution is inherently highly imbalanced. Therefore we employed an ensemble approach by repeated random sampling from the negative training examples 10,000 times, each time matching the number of positive training examples. This ensemble of balanced base classifiers incorporates diverse aspects of negative examples while retaining the sensitivity to positive examples. The final predictions were made by additively combining the prediction probabilities from all base classifiers.

The fold ratios for each phosphorylation site over the 9 time points were scaled between [0, 1] and area under the curve (AUC) (Figure S4B) and polynomial curve fitting (order = 2) was performed for each phosphorylation site and these were used as descriptive features for the SVM. Other features used for SVM training were the average fold ratios for each site across all time points, the fold ratios with insulin and insulin +PI3K or Akt inhibitors, and the position-specific scoring matrix of amino acids surrounding the phosphorylation site (sequence window 13 amino acids).

To determine if the prediction score was substantially higher for predicted kinase than the other kinases, we calculated a 'delta score' (k_{delta}) for each phosphorylation site by subtracting the score received by classification for the next highest kinase ($k_{npredict}$) from the prediction score ($k_{predict}$):

$$k_{delta} = k_{predict} - k_{npredict}$$

A Pareto ranking approach was subsequently used to objectively incorporate both ensemble prediction scores and delta scores for prioritising potential Akt, mTOR and PKA substrates for future follow up molecular characterisation (Figure S5A).

Ensemble prediction performance was estimated by stratified 10-fold cross validation to form partitioned testing and training data sets.

$$Sensitivity = \frac{TP}{(TP + FN)}$$

$$Specificity = \frac{TN}{(TN + FP)}$$

Since it is not known which substrates are true positives other than the manually curated substrates we treated all phosphorylation sites not included in the curated positive substrates as negatives. Assuming that other positive substrates have similar prediction scores as those of curated substrates, this enables us to estimate sensitivity and a lower bound of specificity (Figure S5C).

mTORC2 In Vitro Kinase Assay

Cells at 75-80% confluency were serum starved overnight, stimulated with insulin (100 nM) for 10 minutes, rinsed with ice-cold PBS and lysed in CHAPS IP buffer (40 mM HEPES pH 7.5, 120 mM NaCl, 1 mM

EDTA, 0.3% CHAPS, 10 mM Na-pyrophosphate, 10 mM β-glycerophosphate, 50 mM NaF, Complete EDTA-free protease inhibitors) with rotation at 4°C for 15 minutes, and spun at 16,000 xg for 15 minutes. Protein content was determined by Bradfords assay and samples diluted in CHAPS IP buffer to 1 mg/mL. 4 uL anti-Rictor antibody was added to each sample and incubated with gentle rotation for 90 minutes at 4°C, followed by 15 uL protein G-sepharose for a further 60 minutes. Immunoprecipitates were were washed 4 times with CHAPS IP buffer, and 1 time with kinase buffer (25 mM HEPES pH 7.5, 100 mM potassium acetate, 1 mM $MgCl_2$). After washing the beads were dried and 45uL kinase buffer was added to each containing inactive Akt (500 ng) and 500 uM ATP. Kinase reaction was performed with mixing at 37°C for 30 minutes, terminated immediately by the addition of 2x SDS sample buffer and analysed by immunoblotting.

Akt In Vitro Kinase Assay

HEK293 cells were transiently transfected with HA-SIN1 wiltype or HA-SIN1 T86A. Cells were serum starved overnight, harvested in RIPA buffer (40 mM HEPES pH 7.5, 150 mM NaCl, 2 mM EDTA, 1% Sodium Deoxycholate, 1% NP40, 0.1% SDS, 10 mM Na-pyrophosphate, 10 mM β-glycerophosphate, 50 mM NaF, Complete EDTA-free protease inhibitors), lysed for 20 minutes and centrifuged at 16,000 xg for 15 minutes at 4°C. Protein content was determined by BCA assay and samples diluted to 1 mg/mL. 20 uL anti-HA-sepharose was added to 1 mg protein and immunoprecipitated at 4°C for 3 hours with gentle rotation. Immunoprecipitates were washed 4x with RIPA buffer, and 1x with kinase buffer. After washing the beads were dried and 45uL kinase buffer was added to each containing active Akt (200 ng) and 250 uM ATP. Kinase reaction was performed with mixing at 37°C for 30 minutes, terminated immediately by the addition of 2x SDS sample buffer and analysed by immunoblotting.

Supplemental References

Ben-Hur, A., Ong, C.S., Sonnenburg, S., Scholkopf, B., and Ratsch, G. (2008). Support vector machines and kernels for computational biology. PLoS Comput Biol *4*.

Cox, J., and Mann, M. (2008). MaxQuant enables high peptide identification rates, individualized p.p.b.-range mass accuracies and proteome-wide protein quantification. Nat Biotechnol *26*, 1367-1372.

Cox, J., Neuhauser, N., Michalski, A., Scheltema, R.A., Olsen, J.V., and Mann, M. (2011). Andromeda: a peptide search engine integrated into the MaxQuant environment. J Proteome Res *10*, 1794-1805.

Huttlin, E.L., Jedrychowski, M.P., Elias, J.E., Goswami, T., Rad, R., Beausoleil, S.A., Villen, J., Haas, W., Sowa, M.E., and Gygi, S.P. (2010). A tissue-specific atlas of mouse protein phosphorylation and expression. Cell *143*, 1174-1189.

Larsen, M.R., Thingholm, T.E., Jensen, O.N., Roepstorff, P., and Jorgensen, T.J.D. (2005). Highly selective enrichment of phosphorylated peptides from peptide mixtures using titanium dioxide microcolumns. Mol Cell Proteomics *4*, 873-886.

Olsen, J.V., Blagoev, B., Gnad, F., Macek, B., Kumar, C., Mortensen, P., and Mann, M. (2006). Global, in vivo, and site-specific phosphorylation dynamics in signaling networks. Cell *127*, 635-648.

Ong, S.-E., and Mann, M. (2006). A practical recipe for stable isotope labeling by amino acids in cell culture (SILAC). Nat Protoc *1*, 2650-2660.

Schwanhausser, B., Busse, D., Li, N., Dittmar, G., Schuchhardt, J., Wolf, J., Chen, W., and Selbach, M. (2011). Global quantification of mammalian gene expression control. Nature *473*, 337-342.

Wisniewski, J.R., Zougman, A., and Mann, M. (2009). Combination of FASP and StageTip-based fractionation allows in-depth analysis of the hippocampal membrane proteome. J Proteome Res *8*, 5674-5678.
